# Mind the (expectation) gap: the double bind for women GPs

**DOI:** 10.3399/bjgp25X741081

**Published:** 2025-03-28

**Authors:** Ruth Abrams

**Affiliations:** Senior Lecturer in Workforce, Organisation and Wellbeing, Department of Health Sciences, University of Surrey, Guildford.

## Women GPs and the workforce crisis

We need to talk about women GPs. While we know that experiences of burnout, stress, and anxiety are currently affecting both men and women in general practice, women GPs encounter both internal and external pressures that are unique to them. For example, women GPs are more likely to juggle family responsibilities in addition to their work. Physiologically they will experience at least one of the following: menstruation, menopause, childbirth, infertility, miscarriage, and/or stillbirth. They also encounter limited mentoring, and a lack of leadership opportunities, as well as one of the worst pay gaps in medicine.^1^^,^^2^ Women GPs are often sought out by both patients and colleagues to take on work that demands more emotional labour.^3^^,^^4^ They are also given work that may lead to an increase in workload including women’s health and children’s appointments, and/ or supporting teams.^5^ These gendered pressures have the potential to contribute to and exacerbate their mental and physical ill health, as well as their success at work. Unsurprisingly, women GPs experience more emotional exhaustion at work, higher rates of burnout, stress, and anxiety,^2^ and greater job strain.^6^

## The double bind, double disadvantage

In general practice, women GPs encounter a complicated double bind. They are required to exhibit stereotypically female traits of deference, including empathy, warmth, approachability, likeability, and nurturance. At the same time, they are required to embody stereotypically masculine traits of dominance including assertively enforcing strict 10-minute appointments and aspirations/attainment of partnership roles/leadership positions. In addition to this double bind, a myriad of gendered social norms affects a woman GP’s ability to bridge the expectation gap, including high childcare costs for those with children, cultural expectations in certain demographic groups, and overt sexism among colleagues and patients directed at women GPs.^5^ Women GPs with other intersecting demographic characteristics also encounter a ‘double disadvantage’, whereby gender, combined with, for example, ethnicity, mean that these women have to work twice as hard to overcome the cultural assumption that the ideal GP is a man, white, and UK-trained.^1^

There is a rich landscape in which feminist scholars have theorised double binds affecting women across a range of workplace settings. Unique to our neoliberal society are the ways in which these double binds have become particularly harmful. Often referred to as postfeminism or neoliberal feminism,^7^ double binds now encourage women to absorb and internalise inequalities. Double binds set a powerful cultural scene for the ideal female role model, albeit one who takes sole responsibility for their wellbeing and career success. This makes the bind an individual responsibility, rather than something to be collectivised around and dismantled. Thus, when women encounter a gender pay gap, bullying and harassment, and/ or unsustainable workplace demands, the current social expectation encourages a focus on what an individual can do to overcome these issues. This may look like ‘leaning in’ in order to access a seat at the table;^8^ working on the way they look and act in order to secure positions within the workplace through confidence;^9^ individually negotiating their salary;^10^ and having copious amounts of personal resilience in reserve, to better bounce-back in times of adversity (and austerity).^11^

This form of ‘hyperactive femininity’ is geared towards individualised self-work designed to hide challenges, insecurities, and barriers beyond individual control.^12^ In ‘doing’ this self-work, women oscillate between empowerment and success on the one hand, and disempowerment and perceived failure on the other.^10^ This rise in self-work has, however, also been linked to a rise in individual anxiety, stress, depression, and feelings of insecurity.^13^ Indeed, an international qualitative study into women GPs’ resilience identified that competing demands and gendered expectations created an environment whereby women GPs internalise expectations of themselves, leading to the individualisation of systemic issues and feelings of guilt.^14^

## What’s next for women GPs?

This paints a picture of doom and gloom. It appears that a double bind and a double disadvantage means that women GPs may experience only partial success and, arguably, partial wellbeing in the current set up of general practice. Moreover, this appears to only be possible when conforming to a masculine career model.^1^ However, there is also a glimmer of hope. The tides are turning and attention on supporting the workforce is gaining traction. We see this in funding calls specifically made available for workforce research in and of its own right.^15^

We need this research to draw on new frames of reference and theories that take a critical approach, to prevent further individualisation of systemic issues. Being sensitive to narratives-in-action via empirical data and those informing secondary data will allow us to better understand how the experiences of doing health care shapes women’s thoughts about themselves, their capacities, and responsibilities, as well as the emotional impact of wider social expectations on these women.^10^ We need tailored initiatives that specifically support and address issues women GPs face, above and beyond interventions designed to improve confidence and/ or resilience. We also need spaces for challenging conversations to collectively illuminate contemporary experiences that are assumed to be ‘just the way things are’.

All of this matters because half the medical workforce is now made up of women.^1^ It also matters because of the need to enhance the attractiveness of a career in general practice, for those entering into the profession, for those in it, and even for those retiring from it, all of whom deserve to be well. It matters for the long-term sustainability of general practice. And it matters to and for patient care, and the implications of wider societal expectations.
